# A Novel Pathogenic Mechanism of Highly Pathogenic Avian Influenza H5N1 Viruses Involves Hemagglutinin Mediated Resistance to Serum Innate Inhibitors

**DOI:** 10.1371/journal.pone.0036318

**Published:** 2012-05-01

**Authors:** Jutatip Panaampon, Nathamon Ngaosuwankul, Ornpreya Suptawiwat, Pirom Noisumdaeng, Kantima Sangsiriwut, Bunpote Siridechadilok, Hatairat Lerdsamran, Prasert Auewarakul, Phisanu Pooruk, Pilaipan Puthavathana

**Affiliations:** 1 Department of Microbiology, Faculty of Medicine Siriraj Hospital, Mahidol University, Bangkok, Thailand; 2 Center for Emerging and Neglected Infectious Disease, Mahidol University, Bangkok, Thailand; 3 Department of Microbiology and Immunology, Faculty of Tropical Medicine, Mahidol University, Bangkok, Thailand; 4 Department of Preventive Medicine, Faculty of Medicine Siriraj Hospital, Mahidol University, Bangkok, Thailand; 5 National Center for Genetic Engineering and Biotechnology, Pathumthani, Thailand; Centre of Influenza Research, The University of Hong Kong, Hong Kong

## Abstract

In this study, the effect of innate serum inhibitors on influenza virus infection was addressed. Seasonal influenza A(H1N1) and A(H3N2), 2009 pandemic A(H1N1) (H1N1pdm) and highly pathogenic avian influenza (HPAI) A(H5N1) viruses were tested with guinea pig sera negative for antibodies against all of these viruses as evaluated by hemagglutination-inhibition and microneutralization assays. In the presence of serum inhibitors, the infection by each virus was inhibited differently as measured by the amount of viral nucleoprotein produced in Madin-Darby canine kidney cells. The serum inhibitors inhibited seasonal influenza A(H3N2) virus the most, while the effect was less in seasonal influenza A(H1N1) and H1N1pdm viruses. The suppression by serum inhibitors could be reduced by heat inactivation or treatment with receptor destroying enzyme. In contrast, all H5N1 strains tested were resistant to serum inhibitors. To determine which structure (hemagglutinin (HA) and/or neuraminidase (NA)) on the virus particles that provided the resistance, reverse genetics (rg) was applied to construct chimeric recombinant viruses from A/Puerto Rico/8/1934(H1N1) (PR8) plasmid vectors. rgPR8-H5 HA and rgPR8-H5 HANA were resistant to serum inhibitors while rgPR8-H5 NA and PR8 A(H1N1) parental viruses were sensitive, suggesting that HA of HPAI H5N1 viruses bestowed viral resistance to serum inhibition. These results suggested that the ability to resist serum inhibition might enable the viremic H5N1 viruses to disseminate to distal end organs. The present study also analyzed for correlation between susceptibility to serum inhibitors and number of glycosylation sites present on the globular heads of HA and NA. H3N2 viruses, the subtype with highest susceptibility to serum inhibitors, harbored the highest number of glycosylation sites on the HA globular head. However, this positive correlation cannot be drawn for the other influenza subtypes.

## Introduction

Human and H5N1 avian influenza viruses are different in terms of pathogenesis and severity of the disease. While the infections by influenza A(H1N1), A(H3N2) and influenza B viruses are confined mostly to the upper respiratory tract, the infection caused by highly pathogenic avian influenza (HPAI) H5N1 viruses frequently invades lower respiratory tract, induces cytokine storm, and causes severe pneumonia which progresses to acute respiratory distress syndrome and multi-organ failure [1], [2]. Dissemination of H5N1 virus beyond the respiratory tract is well documented. The viral RNA could be detected in the autopsies of several organs [3], cerebrospinal fluid [4], and fetal tissues [5]. In addition, HPAI H5N1 virus could be isolated from a plasma sample of a Thai patient [6]. This information indicates that the propensity to undergo viremic phase is not uncommon for HPAI H5N1 virus infection. On the other hand, there has been just one recent report on viremia in patients infected with the 2009 pandemic A(H1N1) (H1N1pdm) virus [7]. Moreover, viremia associated with seasonal influenza H1N1 and H3N2 viruses is very rare [8]–[10]. These findings indicate that various influenza subtypes are different in the capability to exhibit viremic phase.

Sera and respiratory fluids of mammals contain several innate soluble factors that exhibit anti-influenza activity, for examples, members of the collectin superfamily such as surfactant protein A (SP-A), surfactant protein D (SP-D), conglutinin and mannose-binding lectin (MBL) [11]–[13], member of the pentraxin superfamily such as pentraxin 3 (PTX3) [14], and serum amyloid P component [15]. The binding of human SP-D and MBL to both hemagglutinin (HA) and neuraminidase (NA) can inhibit influenza virus hemagglutinating activity, interfere with virus release through inhibition of viral neuraminidase activity, and hinder viral infection by preventing viral attachment to the cell receptor. The anti-influenza activities of human SP-D and MBL do not involve other complement factors [16]. Interestingly, the highly glycosylated seasonal H1N1 strains are sensitive to inhibition by both SP-D and MBL, while the poorly glycosylated A/Puerto Rico/8/1934(H1N1) (PR8) and H1N1pdm are resistant to either one of them [17]. The long chain pentraxin, PTX3 inhibits several strains of seasonal influenza A(H1N1), A(H3N2) and influenza B viruses [14], though the susceptibility to PTX3 may be strain-specific as some seasonal human influenza isolates including PR8 virus and the H1N1pdm viruses are resistant to PTX3 [17]. Complement, the major component of innate immunity, may play anti-influenza activity independently or in adjunct with other components. Activation of the complement system results in virus aggregation, virolysis or opsonization [18]. It can lead to increased vascular permeability and recruitment of phagocytic cells to destroy the pathogens. MBL, together with complement in guinea pig serum, exerted lytic activity on influenza virus infected-BHK-21 cells through the classical pathway [19]. MBL also binds influenza HA and activates complement through lectin pathway [20]. Moreover, complement together with natural IgM antibody can destroy virus through the classical pathway [21].

In the other system, non-specific inhibitors against influenza viruses present in normal sera of various animal species were classified into three types: α-, β-, and γ-inhibitors based on their chemical composition and several biological properties [22], [23]. α-inhibitors are heat- stable, but receptor destroying enzyme (RDE)-sensitive glycoproteins. It inhibited influenza virus hemagglutination, but not influenza virus infectivity. The examples of α-inhibitors found in serum are soluble mucoproteins. β-inhibitors are non-sialylated, Ca^2+^ dependent and heat-labile but RDE-resistant. Study in bovine and mouse serum suggested that MBL and SP-D belonged to β-inhibitors [24]; and MBL was serologically cross reactive with human mannose-binding protein (MBP) [20]. γ-inhibitors are heat-stable and RDE-sensitive sialylated glycoproteins which compete with cellular receptor for binding with HA; these inhibitors neutralize viral infectivity by blocking the attachment step in the influenza virus replication cycle [23], [25]. In horse, guinea pig and mouse sera, γ-inhibitor had been identified as α2-macroglobulin [25], [26]. In addition, the study in murine model also demonstrated that the α2-macroglobulin could inhibit hemagglutination inhibition (HI) assay, but its activity on microneutralization (microNT) assay against various viruses expressing H3N2 HA was varied among H3N2 isolates [26].

For the influenza viruses to exhibit viremic phase, they should be able to overcome the inhibition by innate serum inhibitors. Therefore, the ability of HPAI H5N1 viruses to spread beyond the respiratory tract is suggestive of their ability to resist the serum inhibitors. Herein, guinea pig sera were used as the model to study serum innate immunity against various influenza viruses of human and avian origins, including seasonal H1N1, H3N2, H1N1pdm, and HPAI H5N1 viruses. The nature of inhibitors was characterized by their sensitivity to heat or RDE treatment. We demonstrated that HPAI H5N1 virus was the only subtype that could resist the inactivation by serum innate inhibitors. Using reverse genetics, the viral resistance to serum inhibitors could be mapped to HA. Our finding suggested a novel pathogenic mechanism on H5N1 virus dissemination beyond the respiratory system.

## Results

### Biological properties of the guinea pig sera

In order to determine role of serum inhibitors against influenza viruses, it is necessary to screen that the test guinea pig sera contained no specific antibodies against the study viruses. Otherwise, it would be difficult to differentiate between inhibitory effects mediated by the serum specific inhibitors and specific antibodies. Sera of three guinea pigs from different batches of animals were screened for specific antibody against all 14 influenza virus isolates and 4 reassorted viruses by HI and microNT assays. The result showed that none of the test sera contained pre-existing HI or NT antibody as screened at the initial serum dilution 1∶10 (data not shown).

It has been known that some serum innate inhibitors are heat-labile and some are heat-stable. There is no control measure to determine stability of those unknown heat-labile components in the stored sera during the on going experiments. Therefore, our study used complement as the representative of heat-labile components based on its abundant amount in guinea pig sera and availability of the measurement method. We assumed that if complement in the test sera did not deteriorate, the other heat-labile components should remain in good condition. Each guinea pig serum was measured for complement titer by complement fixation test using hemolysin sensitized-sheep red blood cells (SRBCs) as the indicator system. We also showed that the three guinea pigs used in our experiments contained comparable serum concentrations of complement, i.e., 32–64 complement hemolytic units; in another word, the serum dilution of 1∶32 or 1∶64 contained one complement hemolytic unit. The native sera and heat-inactivated sera were assayed in parallel for their lytic activity on hemolysin sensitized- SRBCs. The result demonstrated that the native sera completely lysed the sensitized-SRBCs, while the lytic activity was eradicated after heat inactivation.

**Figure 1:**
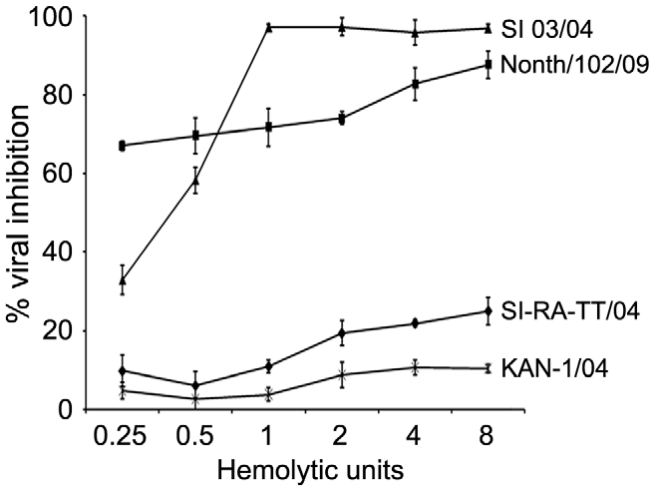
Anti-influenza activities of guinea pig sera at various complement hemolytic units. Native guinea pig sera at various complement hemolytic units were incubated with seasonal H1N1 (SI-RA-TT/04), H3N2 (SI 03/04), H1N1pdm (Nonth/102/09) or HPAI H5N1 (KAN-1/04) virus followed by inoculation the serum-virus mixture onto MDCK cell monolayers. After an overnight incubation, the viral inhibitory activity of the test serum was measured by determining the amount of viral nucleoprotein produced in the inoculated MDCK cells by ELISA. The results are shown as mean of the percentages of viral inhibition derived from quadruplicate experiments. Error bars represent the standard deviation.

Furthermore, the sera were diluted to contain the complement concentrations of 0.25, 0.5, 1, 2, 4 and 8 hemolytic units and assayed against the seasonal A(H1N1) (SI-RA-TT/04), seasonal A(H3N2) (SI 03/04), H1N1pdm (Nonth/102/09) and HPAI H5N1 (KAN-1) viruses in order to determine the optimal dilutions that could exert anti-influenza activity. The result showed a slight increase in the percentages of viral inhibition when serum complement concentrations were greater than two hemolytic units (Figure 1). Therefore, the concentration of 2 complement hemolytic units was employed in subsequent experiments.

### Determination for activity of serum innate inhibitors against various influenza subtypes

**Figure 2:**
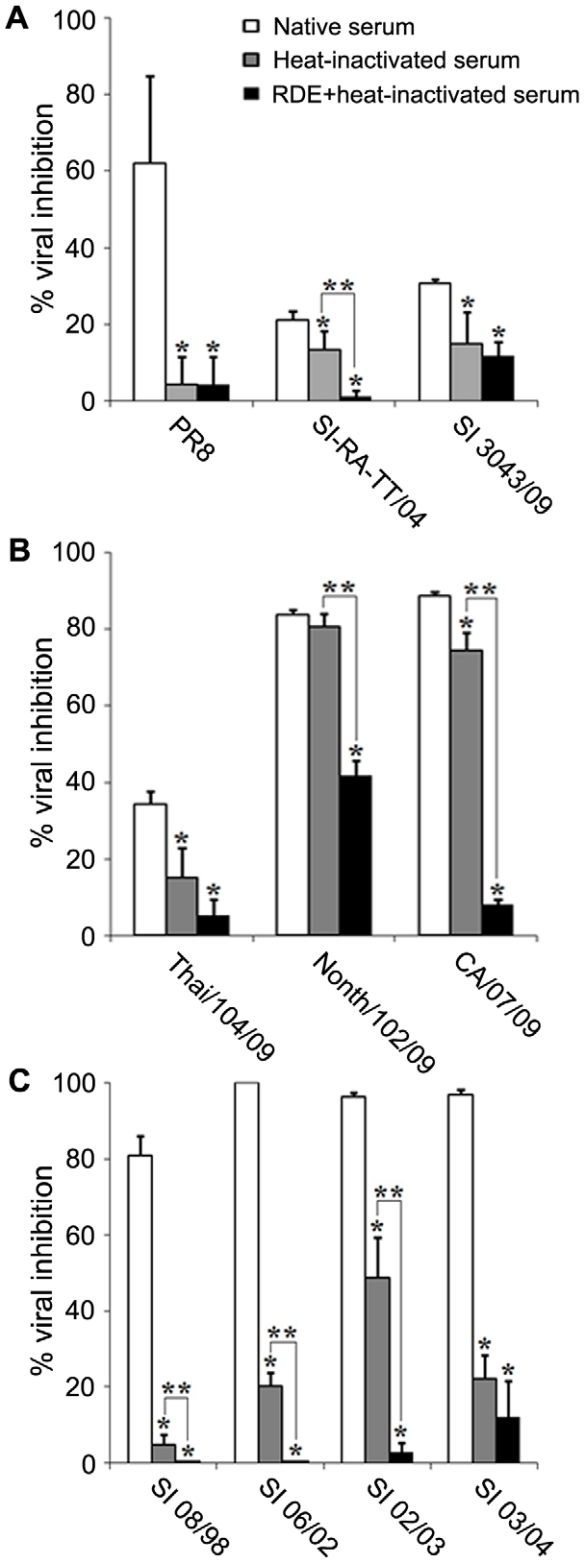
Inhibitory activities of native guinea pig serum, heat-inactivated serum, and RDE-treated+heat-inactivated serum against influenza viruses. Guinea pig sera were incubated with (A) H1N1 viruses, (B) H1N1pdm viruses, or (C) H3N2 viruses. The viral inhibitory activities of the test sera were determined in MDCK cells inoculated with the serum-virus mixtures by ELISA. The results are shown as mean of the percentages of viral inhibition derived from quadruplicate experiments. Error bars represent the standard deviation. * There are statistically significant differences between the inhibitory activity of the native serum and the heat-inactivated serum or the native serum and the RDE-treated+heat-inactivated serum (*p*≤0.05). ** There are statistically significant differences in the inhibitory activity between heat-inactivated serum and RDE-treated+heat-inactivated serum (*p*≤0.05).

Guinea pig sera were used as source of innate inhibitors in the assay for infection inhibition against influenza viruses. The study viruses at concentration of 1,000 TCID50/50 µl (TCID50; 50% tissue culture infectious dose) were incubated with native guinea pig serum at working dilution which contained complement concentration of 2 hemolytic units. The virus-serum mixture in quadruplicate was inoculated onto Madin-Darby canine kidney (MDCK) cell monolayers for overnight. Infectivity of the viruses which were susceptible to serum innate inhibitors should be inhibited and resulted in the decreased amount of viral nucleoprotein produced in the infected MDCK cells as determined by enzyme-linked immunosorbent assay (ELISA). Based on the percentages of viral infection inhibition as compared to the virus control, H3N2 viruses were the subtype most susceptible to serum inhibitors (81–100% inhibition); and followed in order by H1N1pdm (34–89% inhibition) and seasonal H1N1 viruses (21–62% inhibition) (Table 1 and Figure 2). In contrast, all HPAI H5N1 viruses were resistant to serum inhibitors with the percentages of inhibition of less than 10, except one serum which exerted 19% inhibition against KAN-1, and 12% inhibition against NBL (feces in origin) viruses (Table 1).

**Table 1:** Presence of inhibitory factors against influenza viruses in guinea pig sera.

			% inhibition of viral infection mediated by guinea pig sera		
Subtype	Virus name	Abbreviations	No. 1	No. 2	No. 3
**Seasonal H1N1**	A/PR/8/34 (reassorted virus)	PR8	47.7	56.9	62.1
	A/Thailand/Siriraj-Rama-TT/04 (A/New Caledonia/20/99-like virus)	SI-RA-TT/04	21.9	23.3	21.0
	A/Thailand/Siriraj3043/09 (A/Brisbane/59/07-like virus)	SI 3043/09	37.9	31.8	30.7
**Seasonal H3N2**	A/Thailand/Siriraj-08/98 (A/Sydney/05/97-like virus)	SI 08/98	82.6	82.2	80.9
	A/Thailand/Siriraj-06/02 (A/Moscow/10/99-like virus)	SI 06/02	88.7	87.0	100
	A/Thailand/Siriraj-02/03 (A/Fujian/411/02-like virus)	SI 02/03	99.5	97.1	96.2
	A/Thailand/Siriraj-03/04 (A/Fujian/411/02-like virus)	SI 03/04	94.9	97.2	96.9
**H1N1pdm**	A/Thailand/104/09	Thai/104/09	51.6	65.0	34.2
	A/Nonthaburi/102/09	Nonth/102/09	81.2	80.8	83.7
	A/California/07/09	CA/07/09	86.7	85.3	88.7
**HPAI H5N1**	A/Thailand/1(KAN-1)/04	KAN-1	8.9	18.6	5.5
	A/Thailand/676(NYK)/05	676(NYK)	0	2.9	6.0
	A/Thailand/NBL-1/06 (feces)	NBL feces	6.8	12.0	9.8
	A/Thailand/NBL-1/06 (lung)	NBL lung	7.3	6.5	5.2
	A/Laos/Nong Khai 1/07	Nong Khai 1	3.4	7.6	0.8
**H5 reverse genetics**	rgPR8-H5 HANA (KAN-1)	rgPR8-H5 HANA	1.9	7.1	6.4
	rgPR8-H5 HA (KAN-1)	rgPR8-H5 HA	0	4.6	1.6
	rgPR8-H5 NA (KAN-1)	rgPR8-H5 NA	80.0	41.6	84.9

### Biochemical nature of serum innate inhibitors and the viral susceptibility

Heat-inactivated serum or RDE-treated+heat-inactivated serum was assayed for their viral inhibitory effect against various influenza subtypes/strains in comparison with the native serum control. Statistically significant difference in the percentage of inhibition was considered at *p*≤0.05. Each serum was assayed in quadruplicate and the percentages of viral inhibition of all 3 guinea pig sera were averaged. The viral inhibition effects exerted by native sera, heat-inactivated sera and RDE-treated+heat-inactivated sera are shown as the histograms in Figure 2.

Among H1N1 viruses (Figure 2A), PR8 virus was the most susceptible to serum inhibitors (62% viral inhibition) and followed in order by SI 3043/09 and SI-RA-TT/04. Nevertheless, the inhibitory activity against PR8 decreased to almost 4% when the viruses were treated with either heat-inactivated sera or RDE-treated+heat-inactivated sera. Thus, the PR8 virus was susceptible to heat-sensitive inhibitors only, as the addition of RDE (in the RDE-treated+heat-inactivated sera) did not further decrease the percentages of viral inhibitory activity. Similarly, SI 3043/09 was also inhibited by native serum; and percentages of viral inhibition decreased from 31% to 15% with heat-inactivated sera, and to 12% with RDE-treated+heat-inactivated sera. As the decrease in the percentages of inhibition from 15 to 12% was not statistically significant, it is interpreted that SI 3043/09 was susceptible to heat-labile inhibitors, but susceptibility to RDE-sensitive inhibitors, if any, should be very minute. On the other hand, SI-RA-TT/04 was susceptible to both heat-sensitive inhibitors and RDE-sensitive inhibitors as the percentages of viral inhibition dropped from 21% to 13% or 1% after treatment with heat-inactivated sera or RDE-treated+inactivated sera, respectively.

Among H1N1pdm (Figure 2B), percentages of inhibition of Thai/104/09 virus as assayed against native sera, heat-inactivated sera and RDE-treated+heat-inactivated sera were 34, 15 and 5%, respectively. This result showed that Thai/104/09 virus was susceptible to heat-labile serum inhibitors; however, its susceptibility to RDE-sensitive inhibitors could not be drawn statistically. On the other hand, percentages of inhibition of Nonth/102/09 virus as assayed with native sera, heat-inactivated sera and RDE-treated+heat-inactivated sera were 84, 81 and 42%, respectively. The data suggested that Nonth/102/09 virus was partially susceptible to RDE-sensitive inhibitors. The percentages of viral inhibition of CA/07/09 virus as assayed with native sera, heat-inactivated sera and RDE-treated+heat-inactivated were 89, 74, and 8%, respectively. The result suggested that CA/07/09 virus was slightly susceptible to heat-labile inhibitors, but highly susceptible to RDE-sensitive inhibitors.

Interestingly, all H3N2 viruses were highly susceptible to native serum with percentages of viral inhibition greater than 80% (Figure 2C). SI 08/98, SI 06/02 and SI 02/03 were susceptible to both heat-labile inhibitors and RDE-sensitive inhibitors. Such that the percentages of viral inhibition dropped from 81 to 5% for SI 08/98, from 100 to 20% for SI 06/02, and from 96 to 49% for SI 02/03, as the results obtained from the assays with native sera were compared with those employed heat-inactivated sera; and also, the percentages of viral inhibition dropped from 5 to 0% for SI 08/98, from 20 to 0% for SI 06/02, and from 49 to 3% for SI 02/03, as the results obtained from the assay with heat-inactivated sera were compared with those employed RDE-treated+heat-inactivated sera. The statistical analyses were significantly different for those comparisons. On the other hand, SI 03/04 was susceptible to heat-labile inhibitors alone as shown by the significant decrease in the percentages of viral inhibition from 97 to 22% as the assays with native sera were compared with those employed heat-inactivated sera. In contrast, the percentages of viral inhibition which decreased from 22% (as assayed with heat-inactivated sera) to 12% (as assayed with RDE-treated+heat-inactivated sera) was not significantly different.

Owing to low susceptibility of the HPAI H5N1 viruses to native guinea pig sera (the mean percentage of viral inhibition of 11, 3, 10, 6, and 4 for KAN-1, 676(NYK), NBL feces, NBL lung, and Nong Khai 1, respectively), the assays employing heat-inactivated serum as well as RDE-treated+heat-inactivated serum were not performed.

### Role of HA on viral resistance to serum innate inhibitors

**Figure 3:**
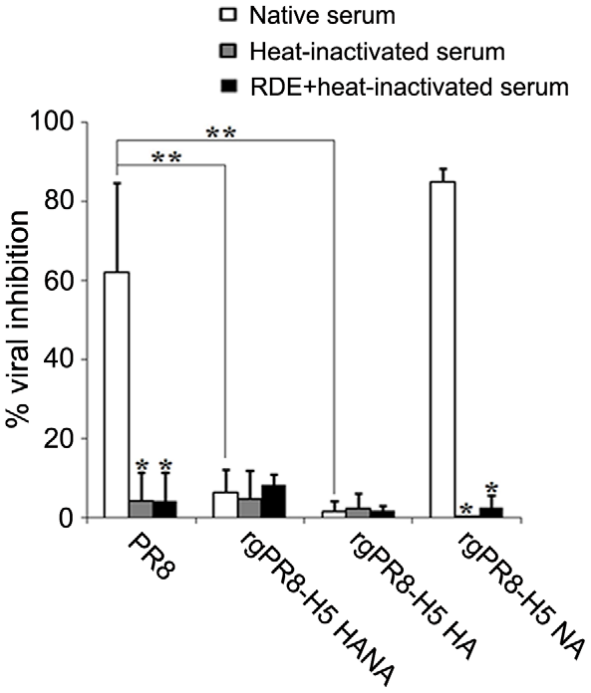
Inhibitory activities of native guinea-pig serum, heat-inactivated serum, and RDE-treated+heat-inactivated serum against reverse genetic viruses. Guinea pig sera were incubated with rgPR8 viruses that contain HPAI H5N1 HANA, HA alone or NA alone. The viral inhibitory activities of the test sera were determined in MDCK cells inoculated with the serum-virus mixtures by ELISA. The results are shown as mean of the percentages of viral inhibition derived from quadruplicate experiments. Error bars represent the standard deviation. * There are statistically significant differences between the inhibitory activity of the native serum and the heat-inactivated serum or the native serum and the RDE-treated+heat-inactivated serum (*p*≤0.05). ** There are statistically significant differences in the serum inhibitory activity as compared between the reassorted PR8 virus and the rgPR8-H5 HA virus or the reassorted PR8 virus and the rgPR8-H5 HANA (*p*≤0.05).

Owing to the difference in susceptibility to serum innate inhibitors among various influenza subtypes, it is speculated that HA and/or NA play a key role in this difference. To elucidate this speculation, reassorted PR8 virus together with three reverse genetic viruses harboring *HA* and/or *NA* genes from HPAI H5N1 (KAN-1) viruses in PR8 backbone, i.e., rgPR8-H5 HANA (6+2 virus), rgPR8-H5 HA (7+1 virus), and rgPR8-H5 NA (7+1 virus) were tested against guinea pig sera. The result showed that reassorted PR8 parental virus and rgPR8-H5 NA could be inhibited by serum innate inhibitors with 48–62% and 42–85% viral inhibition, respectively (Table 1). In contrast, rgPR8-H5 HA and rgPR8-H5 HANA could resist the serum inhibitors with percentages of viral inhibition of only 0–5% for rgPR8-H5 HA and 2–7% for rgPR8-H5 HANA, similarly to the H5N1 wild type viruses. When the viral inhibitory activity of native serum, heat-inactivated serum and RDE-treated+heat-inactivated serum were compared, the result showed that efficiency to inhibit reassorted PR8 parental virus and rgPR8-H5 NA viruses were almost completely lost simply by heat inactivation alone; and thus, the inhibitory effect from RDE could not be seen (Figure 3). No significant change in percentages of viral inhibition was observed with rgPR8-H5 HA and rgPR8-H5 HANA viruses when reacted with either heat-inactivated serum or RDE-treated+heat-inactivated serum. The results were reproducible with all three guinea pig serum samples tested (Table 1). In conclusion, it was the HA not NA that rendered HPAI H5N1 viruses the resistance to serum inhibitors.

### Prediction of the potential N-linked glycosylation sites on HA and NA globular domains

Our previous experiments demonstrated that HA determined the viral susceptibility or resistance to serum innate inhibitors. Additionally, previous groups of investigators reported that an increase in glycosylation site in HA protein resulted in the increased susceptibility to innate inhibitory factors such as SP-D and collectins [27], [28]. Therefore, our study viruses were predicted for their potential sites of glycosylation in HA and NA molecules by CountGS application in BioEdit program version 7.0.4.1 [29]. HA and NA sequences of the study viruses are retrieved from the GenBank database using the accession numbers as shown in Table 2. The potential N-linked glycosylation motif was defined as Asn-X-Ser/Thr, where X may represent any amino acids except proline. The present study determined number of glycosylation sites in the HA globular head of the study viruses, defining that the globular head is situated between the loop region flanked with a conserved disulfide bond in the cysteine bridge in HA1 domain [30]. With methionine as the start position, the globular heads are situated between amino acid position 59 to 291 (reassorted PR8 and seasonal H1N1 viruses), 59 to 292 (H1N1pdm viruses), 68 to 293 (seasonal H3N2 viruses), and 58 to 290 (HPAI H5N1 viruses).

**Table 2:** Potential glycosylation sites on HA and NA proteins of the study viruses.

	Accession number		Number of glycosylation sites on			
Virus name	*HA*	*NA*	HA	Globular head of HA	NA	Globular head of NA
A/PR/8/34 (reassorted virus H1N1)	EF467821	EF467823	7	1	4	2
A/Thailand/Siriraj-Rama-TT/04 (H1N1)	JN676132	JN676133	10	4	9	5
A/Thailand/Siriraj3043/09 (H1N1)	JN676130	JN676131	10	4	9	5
A/Thailand/104/09 (H1N1)	GQ169382	GQ169381	8	1	8	4
A/Nonthaburi/102/09 (H1N1)	GQ150342	GQ150343	8	1	8	4
A/California/07/09 (H1N1)	FJ969540	HM138502	8	1	8	4
A/Thailand/Siriraj-08/98 (H3N2)	JN617980	JN617985	11	6	8	6
A/Thailand/Siriraj-06/02 (H3N2)	JN617982	JN617984	12	7	9	7
A/Thailand/Siriraj-02/03 (H3N2)	JN617979	JN617983	12	7	8	6
A/Thailand/Siriraj-03/04 (H3N2)	JN617981	JN617986	12	7	8	6
A/Thailand/1(KAN-1)/04 (H5N1)	AY555150	AY555151	8	2	3	2
A/Thailand/676(NYK)/05 (H5N1)	DQ360835	DQ360836	8	2	4	3
A/Thailand/NBL1/06 (H5N1) lung	GQ466176	GQ466177	8	2	3	2
A/Laos/Nong Khai 1/07 (H5N1)	EU499372	EU499378	8	2	4	3
rgPR8-H5 HANA (KAN-1)	AY555150	AY555151	8	2	3	2
rgPR8-H5 HA (KAN-1)	AY555150	-	8	2	4	2
rgPR8-H5 NA (KAN-1)	-	AY555151	7	1	3	2

**Figure 4:**
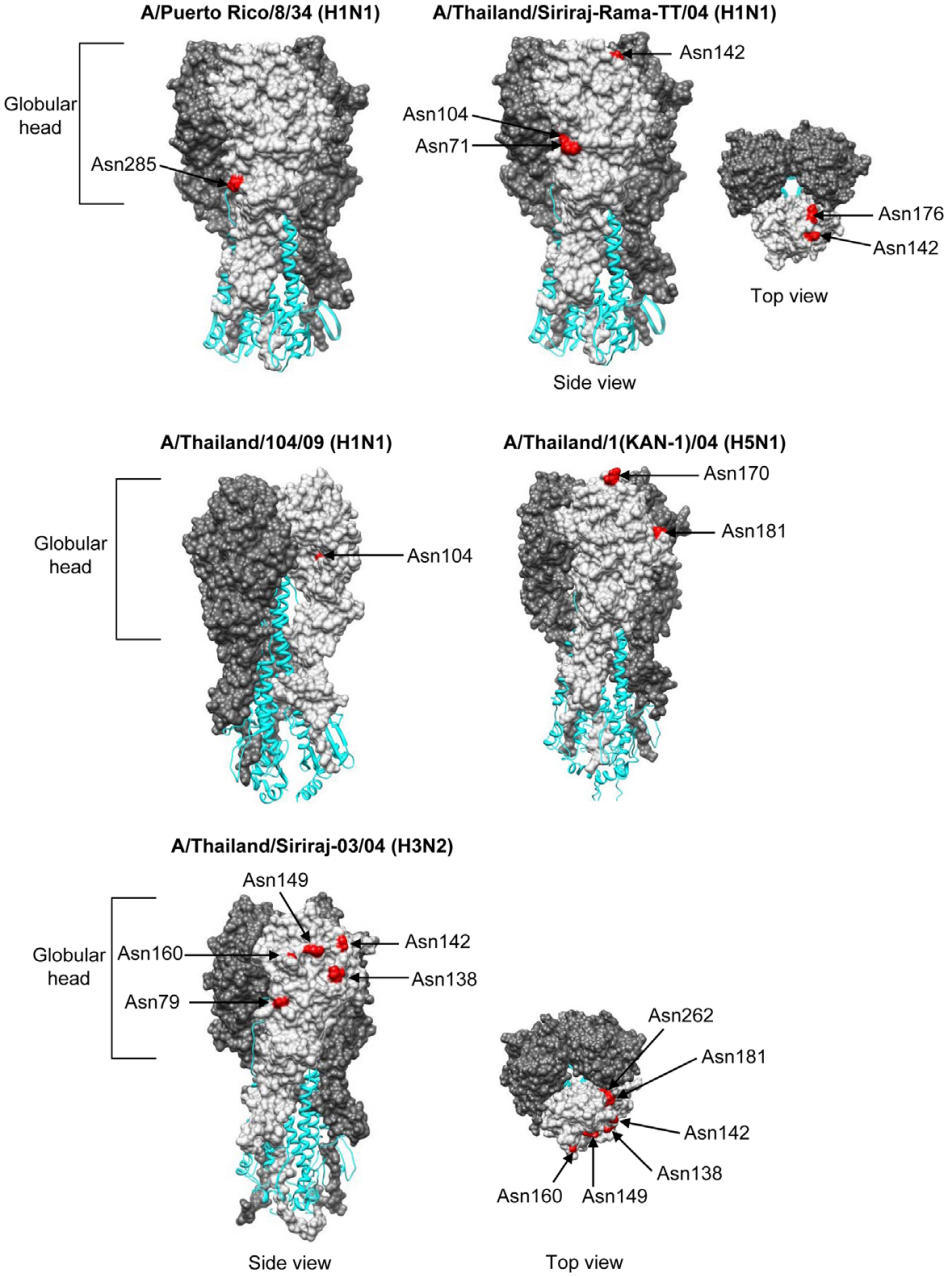
Potential *N*-linked glycosylation sites on HA globular domain. HA1 chains are displayed as space-filling models in HA trimer, while HA2 chains are displayed as ribbon. One of the three HA1 chains is highlighted in lighter shade than the other two HA1 chains. Model of H1N1pdm is related to models of PR8 by 60-degree counterclockwise rotation on vertical axis. The top view is related to the side view by 90-degree rotation on horizontal axis.

The results showed that H3N2 viruses, the most susceptible subtype to serum inhibitors, contained highest number of glycosylation sites in HA. On the other hand, reassorted PR8 and H1N1pdm viruses which were moderately susceptible to serum innate inhibitors contained one glycosylation site on the HA globular head, while the resistant H5N1 viruses harbored 2 glycosylation sites (Table 2). A homology-based structural model of the HA molecules of seasonal H1N1 (SI-RA-TT/04), H1N1pdm (Thai/104/09), seasonal H3N2 (SI 03/04) and HPAI H5N1 (KAN-1) viruses were constructed using HA crystal structures of PR8, A/California/04/09, A/X-31 (H3N2) or A/HK/212/03 (H5N1) (PDB codes: 1RU7, 3LZG, 1HGF and 3FKU, respectively) as the template. All three-dimensional models are generated by Chimera program version 1.5.3 [31] as shown in Figure 4. Amino acid positions that are the potential *N*-linked glycosylation sites on globular head of HA were predicted for reasserted PR8 (Asn285), seasonal H1N1 (Asn71, Asn104, Asn142 and Asn176), H1N1pdm (Asn104), seasonal H3N2 (Asn79, Asn138, Asn142, Asn149, Asn160, Asn181 and Asn262, but Asn160 was not found in SI 08/98 (H3N2)), and H5N1 (Asn170 and Asn181).

Number of glycosylation sites on NA of the study viruses is shown in Table 2. Based on NA crystal structures of A/Brevig Mission/1/18 (H1N1) and A/Memphis/31/98 (H3N2) (PDB codes: 3BEQ and 2AEP, respectively) with methionine as the start position, the globular head domain of N1 NA starts at position 83 to 468 (N1 numbering) and that of N2 NA starts at position 82 to 469 (N2 numbering). Amino acid positions that are the potential *N*-linked glycosylation sites on globular head of NA were predicted for reassorted PR8 (Asn88 and Asn235), seasonal H1N1 (Asn88, Asn146, Asn235, Asn434 and Asn455), H1N1pdm (Asn88, Asn146, Asn235 and Asn385), seasonal H3N2 (Asn86, Asn146, Asn200, Asn234, Asn392 and Asn402, but additional site, Asn93, was found in SI 06/02), and H5N1 viruses (Asn126, Asn215, but additional site, Asn321, was found in 676(NYK), and Asn366 was found in Nong Khai 1 virus).

## Discussion

Most of human sera contain pre-existing anti-influenza antibodies as the results of vaccinations or past infections with some influenza subtypes. Therefore, guinea pig sera instead of human sera were used as the model to study innate immunity against influenza viruses in order to avoid interference effect from human specific antibody which might create miss-interpretation in the viral infection inhibition assay. Nevertheless, it has been shown that the serum components such as MBL and SP-D are presented in both guinea pig and human sera [11]–[13]. To some extents and with a careful interpretation, our results from guinea pig should be able to extrapolate to humans. Additionally, to ascertain that the guinea pig sera were also free from anti-influenza antibodies, the sera were tested negative against all study viruses at the screening dilution of 1∶10 by HI and microNT assays.

Our viral infection inhibition assay demonstrated that among 10 human influenza viruses (reassorted PR8, 2 seasonal H1N1, 4 seasonal H3N2 and 3 H1N1pdm viruses) tested, 5 were susceptible to both heat-inactivated sera and RDE-treated+heat-inactivated sera, 4 were susceptible to heat-inactivated sera only and 1 was susceptible to RDE-treated+heat-inactivated sera only, with degree of inhibition varying to virus subtypes and strains within the same subtype. H3N2 subtype was the most susceptible to serum inhibitors, while seasonal H1N1 and H1N1pdm viruses were moderately susceptible. All HPAI H5N1 isolates tested were highly resistant to serum inhibitors. While the identities of the serum inhibitors were not determined in our study, the class of serum innate immunity could be estimated from the viral inhibition effects after the sera were treated with heat or RDE+heat. Since α-inhibitors can inhibit only hemagglutination but not virus infection [22], [23], they are likely not contributing to the virus inhibition observed in our experiments. On the other hand, our results suggested that serum innate inhibitors against the non H5N1 influenza viruses could be β-inhibitors which are heat-labile and RDE-resistant, γ-inhibitors which are heat-stable and RDE-sensitive, and complement which is heat-labile.

While most of investigators utilized individual serum factors such as SP-D, SP-A, MBL or PTX-3 to study influenza innate immunity, serum factors are likely to work together to exert their anti-influenza activities. In addition, there might be several more anti-influenza factors in sera that have not been discovered yet; thus, complicating the interpretation of the results in broader picture of pathogenesis. The activity of individual factor may not represent overall inhibitory activity observed in the native serum. For example, investigators previously reported that PR8 virus was resistant to MBL [17], and SP-D [27]; however, the present study demonstrated that our reassorted PR8 virus was sensitive to native guinea pig sera with 48–62% viral inhibition activity. The effect of reverse genetics manipulation on susceptibility to serum inhibitors has been excluded as our rgPR8-H5 NA was sensitive while rgPR8-H5 HA and rgPR8-H5 HANA were resistant to the serum inhibitors. Therefore, our system of using whole serum to examine anti-influenza innate serum inhibitors provides a suitable platform to dissect how the HPAI H5N1 virus could disseminate to distal organs.

Number of glycosylation sites on the envelope glycoprotein, HA in particular, has been correlated to the susceptibility of influenza viruses to innate serum inhibitors [28]. Our study showed that H3N2, the most susceptible subtype, has the highest number of glycosylation sites on HA as compared to the other virus subtypes, with 11–12 sites on the H3N2 complete HA amino acid sequences and 6–7 sites on the globular head. A previous study demonstrated that high degree of *N*-linked glycosylation could attenuate H3N2 influenza viruses. The addition of oligosaccharide moieties to globular head of HA had been linked to increased sensitivity of H3N2 viruses to SP-D and MBL [32]. Glycosylation sites on HA have accumulated in the human influenza H3N2 virus since its appearance in 1968. This gain of sites and their long-term maintenance are presumed to be due to a selective advantage of glycosylation [33]. Extensive evidences indicated that glycosylation plays important roles in the life cycle of influenza viruses by conferring structural integrity and stability of virus particles as well as modulating the functions of HA and NA in the recognition of host cell receptors, in particular [34]. Mannose-containing oligosaccharides on the virus envelope were shown to be a target for recognition and destruction by lectins of the innate immune system [35]. Based on the data obtained from H3N2 viruses, it is likely that the degree of viral neutralizing activity of our guinea pig sera was positively correlated to number of glycosylation sites present on HA globular head. Nevertheless, our data from other human influenza subtypes does not support this hypothesis. Our reassorted PR8 and H1N1pdm viruses, which are moderately susceptible to serum inhibitors, contain only one glycosylation site on the HA globular head; while HPAI H5N1 viruses, which are the most resistant subtype, contain two sites. These findings suggested that the viral domains other than glycosylation sites on HA globular head could be the target for binding with serum innate inhibitors. In addition, our previous work also suggested that receptor binding site might not be the target of recognition by the serum inhibitors as 676(NYK) which preferred human type receptor (sialic acid-linked α2,6-galactose) was as resistant as the other H5N1 isolates which preferred avian type receptor (sialic acid-linked α2,3-galactose) [36].

Previous studies have shown genetic elements that contribute to the H5N1 viral virulence such as glutamine to lysine substitution at amino acid position 627 in PB2, the presence of multiple basic amino acids at the HA cleavage site, and the presence of a sequence motif, Glu-Ser-Glu-Val at the carboxyl terminus of NS1 protein [37]. Herein, our study suggests a novel mechanism to virulence of HPAI H5N1 viruses through their ability to resist serum innate immunity, enabling the viruses to disseminate beyond the respiratory tract, the primary organ of infection, to various visceral organs. The disseminated viruses may subsequently exert their immunopathological effect via an induction of cytokine storm and multi-organ failure, the hallmarks of HPAI H5N1 in humans.

## Materials and Methods

### Guinea pig sera

Guinea pig blood was purchased from the National Laboratory Animal Center, Mahidol University. When the blood clot retracted, serum was separated by spinning at 2,000 rpm in a refrigerated centrifuge, and then aliquoted and stored at −80°C. Guinea pig sera were negative for antibody to all study influenza viruses as determined by HI and microNT assays.

### The viruses

Influenza A viruses in this study including seasonal H1N1 and H3N2, H1N1pdm and HPAI H5N1 viruses (Table 1), were propagated in MDCK cells (obtained from the American Type Culture Collection; CCL-34). These virus isolates were kindly provided by Siriraj Influenza Cooperative Research Center; and the routine service laboratory, Department of Microbiology, Faculty of Medicine Siriraj Hospital, Mahidol University.

### Construction of influenza reassortants by reverse genetics

The pHW reverse genetic system [38] comprising 8 recombinant plasmids containing *PA*, *PB1*, *PB2*, *NP*, *HA*, *NA*, *M* or *NS* genomic segments derived from the parental PR8 virus were kindly provided by Prof. Robert Webster and Dr. Erich Hoffman, St. Jude Children Research Hospital, Memphis, Tennessee, USA. Three kinds of reverse genetic viruses harboring *HA*, *NA*, or both *HA* and *NA* genes derived from A/Thailand/1(KAN-1)/04 (H5N1) viruses were constructed. *HA* and *NA* genomic segments were amplified by RT-PCR using universal primers [39] and cloned individually into pHW2000 vector. *HA* segment (accession number AY555150) was engineered to eliminate the multiple basic amino acids at HA cleavage site (337-PQRERRRKKR-346 was changed to 337-PQ----IETR-346, H5 numbering) which mimics the cleavage site of the low pathogenic avian H6 virus before cloning; while the *NA* segment (accession number AY555151) was cloned unmodified. To construct the reverse genetic viruses, rgPR8-H5 HA (7+1), rgPR8-H5 NA (7+1) and rgPR8-H5 HANA (6+2) viruses, 1 µg of each of the 8 recombinant plasmids were transfected into HEK-293 cells co-culturing with MDCK cells using *Trans*IT®-LT1 Transfection Reagent (Mirus Bio LLC, Madison, WI). After 24 hours, the reassortants were rescued by adding with 1 ml of Opti-MEM® I Reduced Serum Medium (Invitrogen, Carlsbad, CA) containing 2 µg/ml of TPCK-treated trypsin (Sigma, St. Louis, MO). The culture plate was further incubated at 37°C for 24–48 hours, observed for cytopathic effect and screened for progeny viruses released in the culture supernatant by hemagglutination assay. In addition, all 8 pHW recombinant plasmids containing each gene of PR8 origin were transfected into HEK-293 cells co-culturing with MDCK cells to generate reassorted PR8 virus as the control of the experiments. The reassortants were propagated and titrated in MDCK cells.

### HI assay

HI assay was performed based on the protocol as described in the WHO manual [40]. Goose RBCs were employed in the assay for antibody to HPAI H5N1 and H1N1pdm. On the other hand guinea pig RBCs were employed in the assay for antibody to the other human influenza viruses. Briefly, 50 µl of guinea pig serum was mixed with 150 µl of RDE (Denka Seiken Co. Ltd., Tokyo, Japan) and incubated at 37°C for 16–18 hours to eliminate the nonspecific inhibitors. The treated serum was inactivated at 56°C for 30 minutes and absorbed with the test RBCs to remove nonspecific agglutinators. The replicating influenza virus at the final concentration of 4 hemagglutination units/25 µl was used as the test antigen. The treated serum was serially two-fold diluted at an initial dilution of 1∶10 into a final volume of 25 µl per well before 25 µl of the test antigen was added. After incubation at room temperature for 30 minutes, 50 µl of 0.5% goose RBCs (for HI antibody to H1N1pdm and H5N1 viruses) or 0.75% guinea pig RBCs (for HI antibody to seasonal H1N1 and H3N2 viruses) were added into every well and further incubated the reaction plate at 4°C 30 minutes (for goose RBCs) or 60 minutes (for guinea pig RBCs). HI titer was defined as the reciprocal of the last serum dilution that completely inhibited hemagglutination of RBCs.

### MicroNT assay

Guinea pig sera were screened for the antibodies to the test influenza viruses by microNT assay using the protocol as described previously [40]. Guinea pig serum was treated with RDE before heat inactivation at 56°C for 30 minutes. The treated sera were serially two- fold diluted with 1X EMEM at an initial dilution of 1∶10 in a microtiter plate. The diluted serum was incubated with the test virus at the final concentration of 100 TCID50/100 µl for 2 hours at 37°C. Then, 100 µl of each virus-antibody mixture was transferred onto the MDCK cell monolayer maintained in EMEM supplemented with trypsin TPCK (Sigma, St. Louis, MO), and incubated overnight at 37°C, 5% CO_2_. The inoculated MDCK cell monolayers in the reaction plates were fixed with 80% acetone prior to determining for presence of viral nucleoprotein by ELISA. Antibody titer was defined as the highest serum dilution that yielded a 50% reduction in the amount of nucleoprotein as compared to the virus infected cell control.

### Complement titration

Guinea pig serum contains many soluble factors, including complement factors that confer activity against influenza virus. The amount of complement present in each guinea pig serum can be measured by titration against hemolysin-sensitized SRBCs [41]. Equal volume of 2% SRBCs was mixed with appropriate concentration of hemolysin (Sigma-Aldrich, St. Louis, MO) for 1 hour with shaking at intervals to obtain hemolysin sensitized SRBCs. The test guinea pig serum was serially two-fold diluted with veronal buffer to obtain a volume of 25 µl per well in a U-shaped microtiter plate. Then, the serum was incubated with hemolysin sensitized SRBCs for 45 minutes with shaking at intervals. In the presence of serum complement, the sensitized SRBCs would be lysed; while the SRBCs and sensitized SRBC controls appeared as a red button at the bottom of the well. One hemolytic unit was defined as the highest dilution of complement which yielded complete hemolysis of the sensitized SRBCs. Owing to heat-labile nature of the complement proteins, guinea pig sera were stored at −70°C; and complement titration was conducted prior to each use.

### Assay for inhibition of influenza virus infection by soluble factors in guinea pig serum

Guinea pig serum at the working concentration of 2 complement hemolytic units was incubated with the test virus at a final concentration of 1,000 TCID50/50 µl at 37°C for 45 minutes in quadruplicate wells. Then, the mixtures were transferred onto MDCK cell monolayers and incubated overnight at 37°C prior to measuring the amount of viral nucleoprotein in the infected MDCK cell monolayers by ELISA. In the presence of serum inhibitors, viral infection was blocked, resulting in the decreased amount of viral nucleoprotein synthesized in the inoculated culture as compared to the infected MDCK cell controls. In the case where serum inhibitors could not inhibit viral infection, the high O.D. values indicating viral nucleoprotein synthesis would be observed. The percentages of viral inhibition were calculated based on the O.D. values as compared to the virus control without serum.

The assay employed native guinea pig serum was conducted in parallel with the heat-inactivated serum and together with the RDE-treated+heat-inactivated serum. Heat treatment to inactivate the β-inhibitors was performed by treating the guinea pig serum at 56°C for 30 minutes and then cooled on ice. RDE treatment was conducted to destroy α- and γ-inhibitors such that the guinea pig serum was mixed with RDE according to the protocol used for HI assay, followed by heat inactivation at 56°C for 30 minutes to get rid of RDE activity and β-inhibitors.

### ELISA

ELISA for influenza viral nucleoprotein was performed with mouse specific monoclonal antibody against influenza nucleoprotein (Catalog number MAB8257, Millipore Corporation, Temecula, CA) as the primary antibody. Horseradish peroxidase (HRP)-conjugated goat anti-mouse Ig (Catalog number 1010-05, Southern Biotech, Birmingham, AL) was used as the secondary antibody. The optical density was measured at dual wavelengths of 450/630 in a spectrophotometer.

### Statistical analysis

Statistical analysis was done with PASW Statistics 18 software. The percentages of viral inhibition by native sera, heat-inactivated sera and RDE-treated+heat-inactivated sera were analyzed using analysis of variance (ANOVA). Difference between two data sets was examined using Student's *t*-test. The significance level was set at *p*≤0.05.
